# Reply to: Current Status and Management Strategies of Obstetric Hemorrhage Using Contrast-Enhanced Dynamic Computed Tomography in a Representative Tertiary Perinatal Medical Center in Japan

**DOI:** 10.31662/jmaj.2025-0095

**Published:** 2025-05-30

**Authors:** Yoshitsugu Chigusa, Naohiro Suzuki

**Affiliations:** 1 Department of Gynecology and Obstetrics, Graduate School of Medicine, Kyoto University, Kyoto, Japan

**Keywords:** Obstetric hemorrhage, Contrast-enhanced computed tomography, and Transcatheter arterial embolization

Dear Editor,

We appreciate the thoughtful comments from Dr. Daungsupawong and Dr. Wiwanitkit regarding our study on obstetric hemorrhage management using contrast-enhanced dynamic computed tomography (CE-dCT). We would like to address their key points.

First, we would like to emphasize that our study analyzed all cases of obstetric hemorrhage, not just uterine bleeding. Our cohort included various sources of bleeding such as vaginal hemorrhage, vulvar hematoma, and retroperitoneal bleeding, representing a notably heterogeneous patient population. This comprehensive approach was intentional because it reflects the diverse reality of obstetric hemorrhage encountered in clinical practice.

Regarding the treatment decision-making process, we would like to clarify that our institution consistently applied the standardized management algorithm that is clearly described in our manuscript and illustrated in Figure 2. For uterine bleeding without extravasation, conservative treatment with uterine contractile agents is indicated. In cases of uterine bleeding with extravasation, the location of extravasation and the presence of PRACE, which is characterized by Postpartum hemorrhage, Resistance to treatment, and Arterial Contrast Extravasation on CE-dCT scans guide our choice between balloon tamponade and transcatheter arterial embolization (TAE). Specifically, our protocol typically begins with balloon tamponade as the initial intervention, and if this proves unsuccessful, we promptly proceed to TAE. The systematic implementation of this algorithm produced successful conservative management in 57% of cases, whereas 43% required invasive interventions.

Concerning the statistical analysis, we should clarify that we did not use multivariate regression models in our study, contrary to what was stated in the Letter. Given the retrospective nature of our study and the heterogeneous population analyzed, we appropriately used Mann-Whitney and Fisher's exact tests to compare outcomes in groups.

We agree with the authors' suggestions regarding future research directions. The long-term outcomes of various treatment modalities and the cost-effectiveness of CE-dCT in obstetric hemorrhage management certainly warrant further investigation. However, we believe that the cost-effectiveness of CE-dCT should be evaluated in the context of its manifest benefits: the technique's ability to detect active bleeding locations and guide appropriate therapeutic interventions, which is crucial for maternal lifesaving. We are currently working to expand this research through multi-center collaborations, which will help establish more robust evidence for our treatment protocol and potentially lead to improved standardization of care for patients with obstetric hemorrhage.

Sincerely,

## Article Information

### Conflicts of Interest

None

